# Maternal protein restriction during perinatal life affects lung mechanics and the surfactant system during early postnatal life in female rats

**DOI:** 10.1371/journal.pone.0215611

**Published:** 2019-04-19

**Authors:** Reza Khazaee, Lynda A. McCaig, Cory Yamashita, Daniel B. Hardy, Ruud A. W. Veldhuizen

**Affiliations:** 1 Department of Physiology & Pharmacology, The University of Western Ontario, London, Ontario, Canada; 2 Biotron Research Centre, The University of Western Ontario, London, Ontario, Canada; 3 Lawson Health Research Institute, London, Ontario, Canada; 4 Department of Medicine, The University of Western Ontario, London, Ontario, Canada; 5 Department of Obstetrics & Gynecology, The University of Western Ontario, London, Ontario, Canada; The University of Manchester, UNITED KINGDOM

## Abstract

Limited information is available on how fetal growth retardation (FGR) affects the lung in the neonatal period in males and females. This led us to test the hypothesis that FGR alters lung mechanics and the surfactant system during the neonatal period. To test this hypothesis a model of FGR was utilized in which pregnant rat dams were fed a low protein diet during both the gestation and lactation period. We subsequently analyzed lung mechanics using a FlexiVent ventilator in male and female pups at postnatal day 7 and 21. Lung lavage material was obtained at postnatal day 1, 7 and 21, and was used for analysis of the surfactant system which included measurement of the pool size of surfactant and its subfraction as well as the surface tension reducing ability of the surfactant. The main result of the study was a significantly lower lung compliance and higher tissue elastance which was observed in FGR female offspring at day 21 compared to control offspring. In addition, female LP offspring exhibited lower surfactant pool sizes at postnatal day 1compared to controls. These changes were not observed in the male offspring. It is concluded that FGR has a different impact on pulmonary function and on surfactant in female, as compared to male, offspring.

## Introduction

Epidemiological studies provide strong evidence for the negative impact of fetal growth restriction (FGR) on numerous short and long-term health outcomes of the offspring [1]. Defined as an infant with a birthweight below the 10^th^ percentile for its gestational age, FGR has been shown to contribute to altered neurodevelopment and impaired growth in the immediate neonatal period, as well as chronic diseases such as diabetes, hypertension, and dyslipidemia later in life [2,3]. Both human epidemiological studies and animal models suggest many of these adverse postnatal outcomes can be sex-specific [4–6]. With respect to the lung, FGR has been implicated in poor pulmonary health; for example it has been suggested to be a risk factor for the development of asthma [7].

Advances in our understanding of how FGR may lead to adverse outcomes in the neonate stems from numerous animal studies ranging from sheep to mice, utilizing methodological approaches including placental embolization, calorie restriction, protein restriction and hypoxia [8,9]. These models all result in placental insufficiency and ultimately impact fetal growth and the potential development of disease at various stages of life Interestingly, organ specific sexual dimorphisms has been observed across a spectrum of pathologies within these models [4,5]. While a large proportion of these studies have focused on various metabolic outcomes involving the liver, kidney and adipose tissue among others, relatively few animal studies to date have studied the mechanisms by which FGR impacts postnatal lung function [7].

With respect to previous studies investigating the effect of FGR on pulmonary outcomes, a variety of studies have demonstrated effects on signaling pathways influencing alveolarization [10–12]. It was also previously demonstrated that FGR sheep had unfavorable changes to respiratory parameters such as decreased lung compliance, tidal volume and minute volume by eight weeks in postnatal life [13]. Furthermore, experiments using a rat model of maternal undernutrition showed that FGR animals had decreased alveolar surface area as compared to control up until 42 days of age [14]. However, to date, there have no differences reported with regards to male and females in these studies exploring pulmonary physiological effects of FGR.

One essential component of normal postnatal lung development is a functional pulmonary surfactant system [15]. Pulmonary surfactant, a lipid protein mixture which reduces the surface tension at the alveolar surface, plays an essential role in maintaining lung compliance during respiration [15]. Surfactant has been investigated in the context of FGR, however, the majority of these studies have focused on the mRNA levels of the surfactant associated proteins, SP-A, B, C and D [16–18]. In general, these studies show decrease in expressions of these proteins during development and the immediate postnatal period FGR. These studies imply changes in surfactant due to FGR, but the impact of these expression profiles on surfactant content and surface activity of surfactant, and the potential sex differences in these outcomes, has not been previously assessed.

Based on this information, it was hypothesized that FGR alters lung mechanics and the surfactant system during the neonatal period. This hypothesis was tested in a rat model of FGR induced by maternal protein restriction in which the male and female offspring were examined at postnatal day 1, 7 and 21.

## Methods

### Animal experimentation

All procedures were approved by the Animal Use Subcommittee at the University of Western Ontario (Protocol Number: 2005–009). A total of ten female and three male Wistar rats at breeding age (250 g) were purchased from Charles River (La Salle, St-Constant, Quebec, Canada). Rats were housed in individual cages and allowed to acclimatize to the animal care facility for three weeks on a 12:12 light: dark cycle, during which time they had free access to water and standard chow. To allow for sufficient time for all analyses, pregnancies were staggered to obtain two litters per week. Briefly, after the acclimatization period, female rats were housed with males in the late afternoon. The following morning, impregnation was confirmed by the presence of sperm in the vaginal smear. Upon confirmation of impregnation (gestation day 0), pregnant rats were housed individually and randomized to one of two dietary conditions: a 20% protein (Control, n = 6 litters) or an 8% low protein diet (LP, lower casein, n = 4 litters) [19,20]. The LP diet contained equal fat content and was made isocaloric by the addition of carbohydrates (13% increase in sucrose) (Bio-Serv, Frenchtown, NJ). To avoid the effects of litter size on our outcomes, immediately after birth the litters were culled to 8 pups, the euthanized animals were used for the d1 data as described below. Mothers were kept on the same dietary regimes until postnatal d21. Weights of the male and female offspring were recorded at d1, d7, and d21 after birth.

### Analysis of lung mechanics

Rats at postnatal d7 and d21 were euthanized via intraperitoneal injection (IP) of Sodium pentobarbital overdose (110mg/Kg of body weight [BW]). After performing a tracheostomy and exposing the lungs, animals were connected to the FlexiVent (SCIREQ, Montreal, Quebec, Canada) for *ex vivo* measurements of lung function [21,22]. Following connecting to the FlexiVent, rats were immediately exposed to 1min *ex vivo* mechanical ventilation (Vt = 10ml/kg, RR = 120breath/min, PEEP = 0cm). Prior to measurements, to standardize volume history, deep inflation was applied to the lungs from PEEP value to about the pressure of 30cmH2O. Following the deep inflation, we performed three different perturbations: a snapshot perturbation, a prime-8, and pressure-volume (PV) curve through controlled stepwise increasing pressure [22]. These software-controlled perturbations were performed with 10 seconds intervals of ex vivo ventilation. The snapshot perturbation was applied to measure compliance and resistance of the whole respiratory system. Prime-8 perturbation outcomes were values for proximal airway resistance, tissue elastance, and tissue dampening. Lastly, PV-P curves generated values for curvature (parameter K) of the upper portion of the deflation PV curve.

### Lung lavage

Following FlexiVent measurements, whole-lung bronchoalveolar lavage (BAL) of d7 and d21 offspring were collected through flushing the lungs with four and five aliquots of saline for d7 and d21, respectively. D1 offspring were too small to be assessed for lung mechanics on the flexivent and were therefore directly utilized for BAL collection after euthanasia (Sodium pentobarbital IP overdose [110mg/Kg BW]) and tracheostomy. At each age, lungs were lavaged with four aliquots of saline at volumes sufficient to fill the lung. The total volume of saline administered and recovered was recorded. Subsequently, the lavage samples were centrifuged at 150g for 10min at 4°C to remove the cellular debris, and the an aliquot of supernatant containing the total surfactant was stored at -20°C while the remainder was centrifuged at 40,000g for 15min to separate large aggregate (LA) surfactant subfraction from the supernatant which contained the small aggregates (SA) [23]. The LA pellets were re-suspended in a known volume of saline. Both the resuspended LA and SA were stored in aliquot at -20°C until further analysis.

### Surfactant analysis

Phospholipid contents of total surfactant aliquot as well as that from the LA and SA subfractions was determined following a lipid extraction followed by phosphorous analysis as previously described [23–25]. Total protein content in lavage was measured using a Micro BCA protein assay kit (Pierce, Rockford, Ill., USA) according to manufacturer’s instructions. For biophysical analysis, aliquots of LA were resuspended at a phospholipid concentration of 2mg/ml in buffer containing 140mM NaCl, 2.5mM HEPES, and 1.5mM CaCl2, pH = 7.4. Samples were incubated at 37°C for at least 1hr prior to assessing their surface tension reducing ability using a constrained sessile drop surfactometer (CDS) as previously described [26,27]. Briefly, a droplet of LA sample (~10 μl) was dispensed onto the CDS drop holder. The droplet was allowed to equilibrate for 2 minutes to allow for surfactant absorption. Following absorption, the droplet was exposed to 20 dynamic compression-expansion cycles on the CDS using a computer-controlled stepper motor (LTA-HS actuator, Newport Corporation, Irvine, CA, USA). Compression-expansion cycles were applied at a frequency of 20 cycles per minute and intended compression of 20–25%. During the dynamic compression-expansion cycles, images of the droplet were taken at a rate of 10 frames per second and the recorded images were analyzed with Axisymmetric Drop Shape Analysis software to assess the sample surface tension and the surface area of each picture.

### Statistical analysis

All litters were culled to 8 pups at day 1, leading to an n-value of 13–26 pups at this time-point. For day 7 and day 21, n-values ranged from 4-11pups with each experimental group containing pups from a minimum of 3 different litters. All data are expressed as mean ± standard error of the mean (SEM). All statistical analyses were performed using the GraphPad Prism statistical software (GraphPad Software, Inc., La Jolla, CA., USA). Statistical comparisons among male and female groups were analyzed by two-way ANOVA with postnatal day and diet as independent variables. Subsequent t-tests were performed to assess statistical comparisons between control and LP diets. Probability (p) values of less than 0.05 were considered statistically significant.

## Results

The bodyweight of the offspring at day 1, 7 and 21 is shown in Table 1. The data indicates lower bodyweight in the low protein diet group compared to the control diet animals in both female and male offspring. This difference was significant at both day 1 and 21. The percent difference (~35%) in body weight at Day 21 was the same between male and female offspring.

**Table 1:** Numbers and body weights in grams of control and low protein offspring at d1, d7, and d21.

	d1		d7		d21	
	Control	LP	Control	LP	Control	LP
**Males**	7.0 ± 0.2, n = 16	6.3* ± 0.3, n = 13	21.2 ± 1.2, n = 10	17.2 ± 1.5, n = 6	73.5 ± 2.5, n = 10	50.1* ± 4.0, n = 10
**Females**	6.6 ± 0.2, n = 26	5.8* ± 0.1, n = 19	18.5 ± 1.6, n = 8	15.7 ± 0.7 n = 11	71.4 ± 3.0, n = 10	45.9* ± 4.7, n = 4

Body weights of LP d1 and d21 males and females were significantly lower compared to control groups (* = p<0.05). There was no difference in body weights of LP offspring at d7 compared to controls (p>0.05).

**Fig 1:**
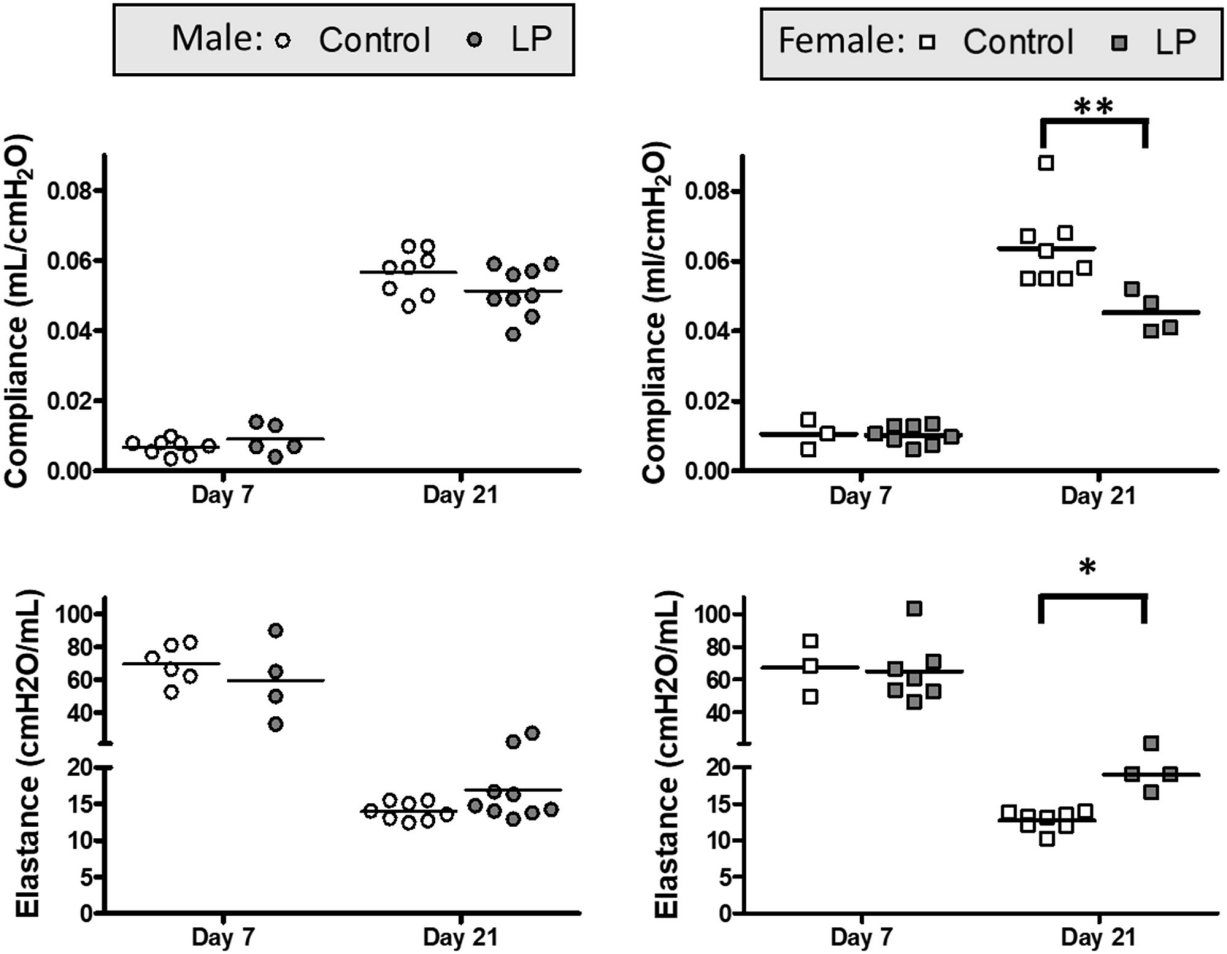
Lung compliance and elastance values. Lung compliance and elastance values in Control and LP males (A, C) and females (B, D) at postnatal d7 and d21 as analyzed on the FlexiVent. * = p < 0.05 and ** = p < 0.01 vs control.

**Fig 2:**
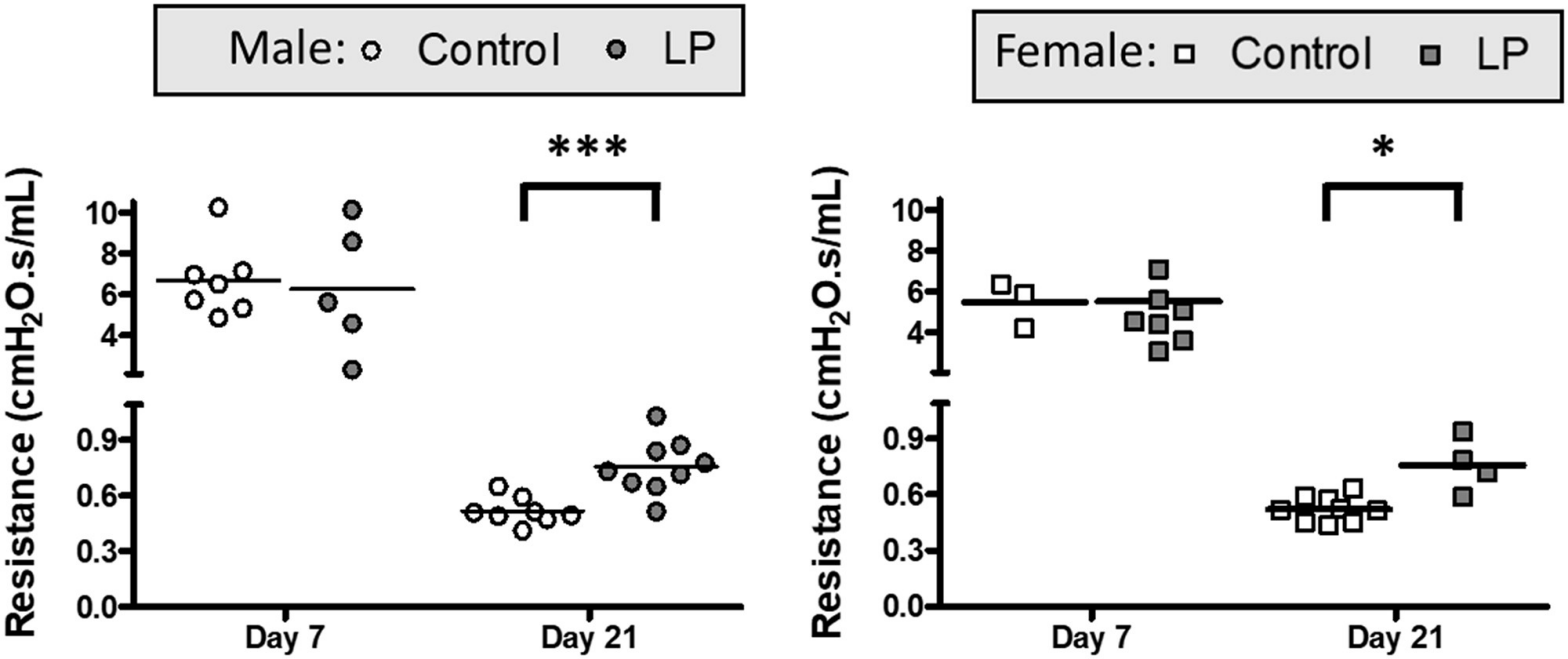
Lung resistance values. Lung resistance values in Control and LP males (A) and females (B) at postnatal d7 and d21 as analyzed on the FlexiVent. * = p < 0.05 and *** = p < 0.001 vs control.

Outcomes related to lung mechanics, assessed for animals at day 7 and 21 using a FlexiVent ventilator, are shown in Figs 1 and 2. Lung compliance increased from day 7 to day 21 in both male and female animals with a concurrent decrease in lung elastance (Fig 1). Comparison of the two dietary groups revealed no significant differences in compliance and elastance in male animals at both day 7 and day 21 (Fig 1A and 1C). In female offspring, while there were no significant differences at day 7, lung compliance at day 21 was significantly lower and elastance significantly higher in the LP group as compared to the control diet (Fig 1B and 1D). Analysis of whole lung resistance revealed an expected decrease associated with growth from day 7 to 21. There was no significant difference between the two diet groups at day 7 for either male or female animals, but at day 21 resistance was significantly higher in the LP group of both sexes compared to control (Fig 2A and 2B).

**Fig 3:**
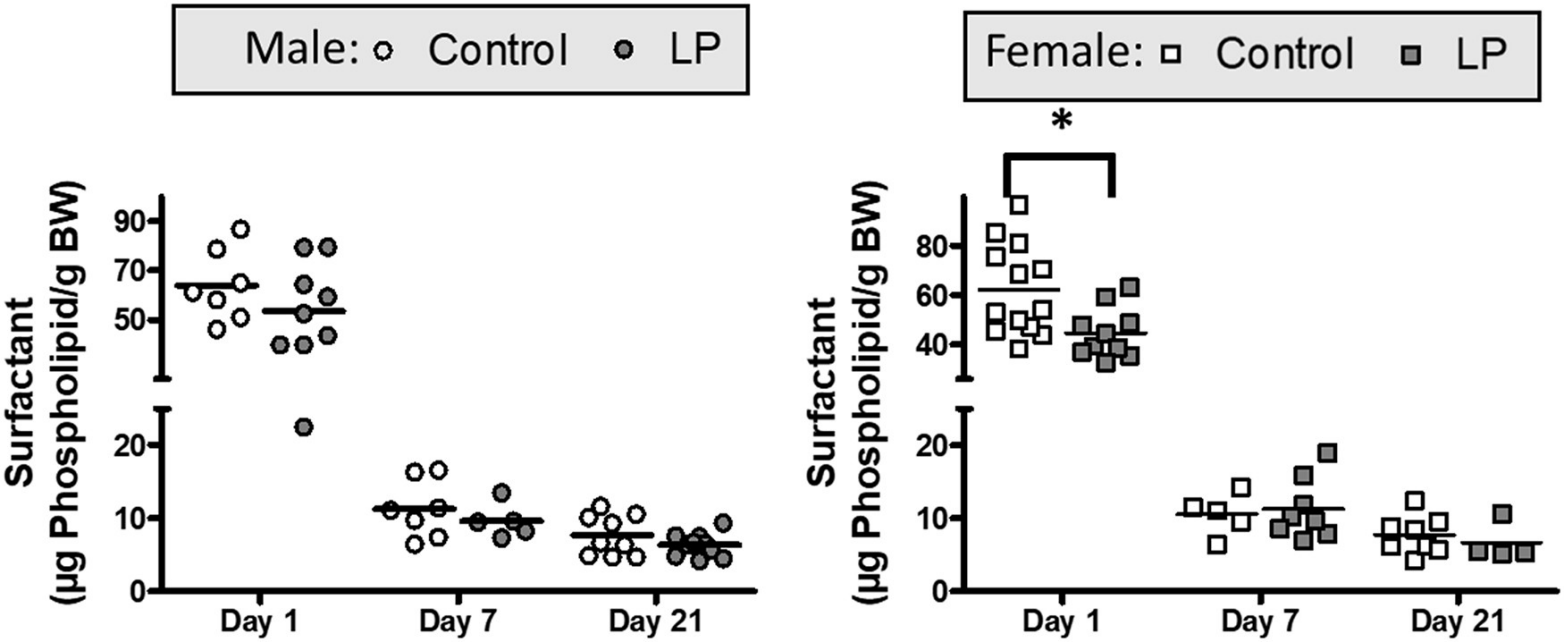
Surfactant values. Total amounts of surfactant recovered from the lung lavage fluid in Control and LP males (A) and females (B) at postnatal d1, d7 and d21 as analyzed via phospholipid-phosphorus analysis. * = p < 0.05 vs control.

Given the alterations in lung mechanics, we next investigated whether alterations in pulmonary surfactant mediated these deficits in lung function. Values of the total surfactant for all three postnatal days are shown in Fig 3. The total amount of surfactant recovered from the lung lavage, expressed relative to bodyweight, was relatively high at postnatal day 1, with lower values recovered at day 7 and 21 for both male and female animals (Fig 3). In male animals, the amount of total surfactant was not significantly different between the two diet groups as each of the three postnatal days examined (Fig 3A). In contrast, in female animals, total surfactant levels were significantly lower in the LP group as compared to controls but only at postnatal day 1 (Fig 3B).

**Fig 4:**
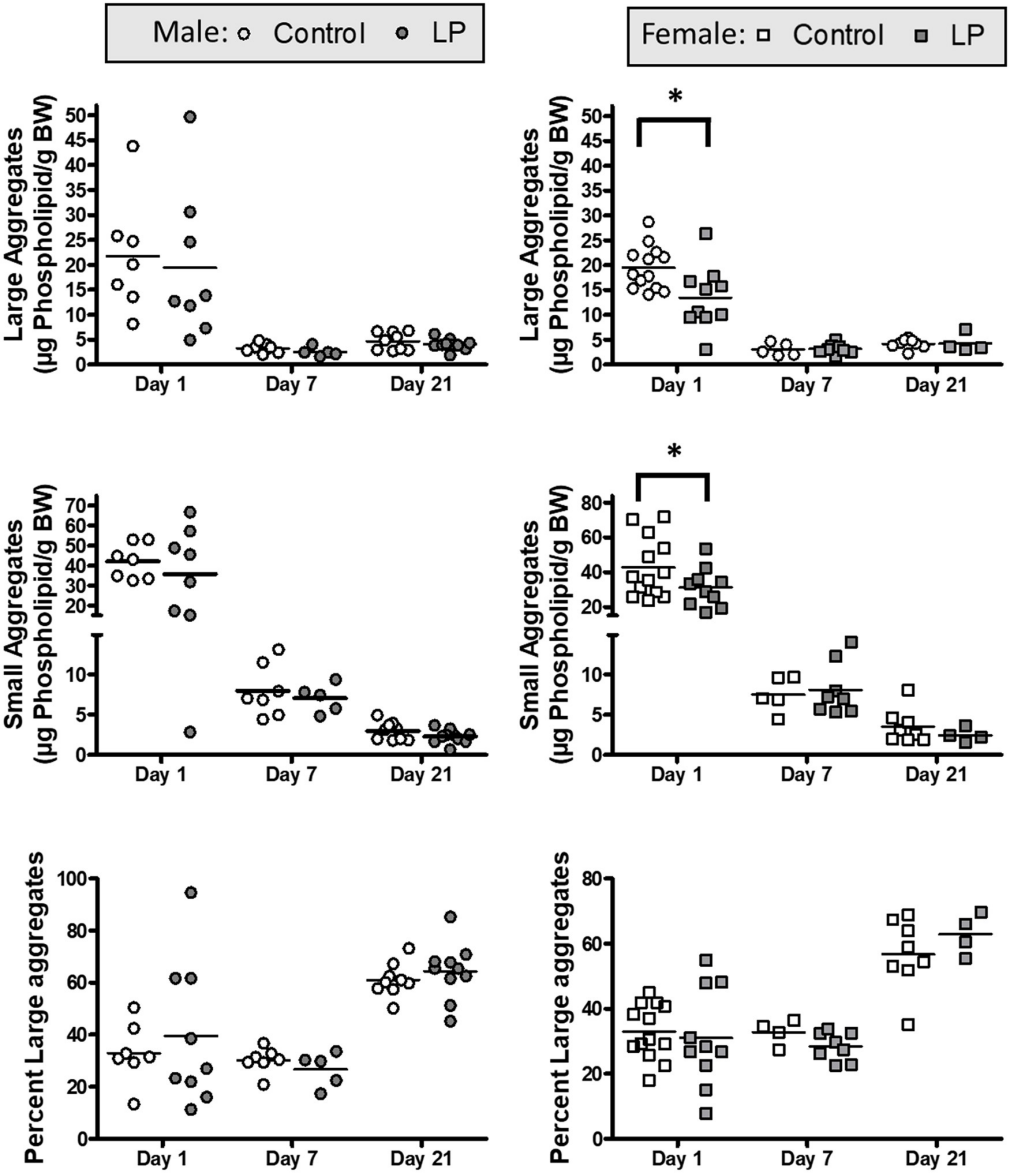
Surfactant subfraction. Amounts of surfactant subfractions, LA and SA, as well as the percentage of LA, recovered from the lung lavage fluid in Control and LP males (A, C, E) and females (B, D, F) postnatal d1, d7 and d21 as analyzed via phospholipid-phosphorus analysis. * = p < 0.05 vs control.

Since total surfactant can be separated into different subfractions, the LA and SA, these pools were analyzed separately and are shown in Fig 4. In both male and female animals, the LA and SA pool sizes decreased from postnatal day 1 to day 7 and 21 (Fig 4A–4D). Expressing the data as percentage of LA demonstrated higher values at day 21 as compared to the two earlier days (Fig 4E and 4F). Comparison between the diet groups revealed no significant differences in any of the postnatal days in the male animals (Fig 4A, 4C and 4E). For female animals both the LA and the SA subfractions were lower in the LP groups at postnatal day 1, with no changes at any of the other days that were analyzed (Fig 4B and 4D). In addition, the lavage fluid was also utilized to measure total protein concentrations, as an indication of protein leak into the lung; there were no significant differences in these values among all groups (data not shown).

**Fig 5:**
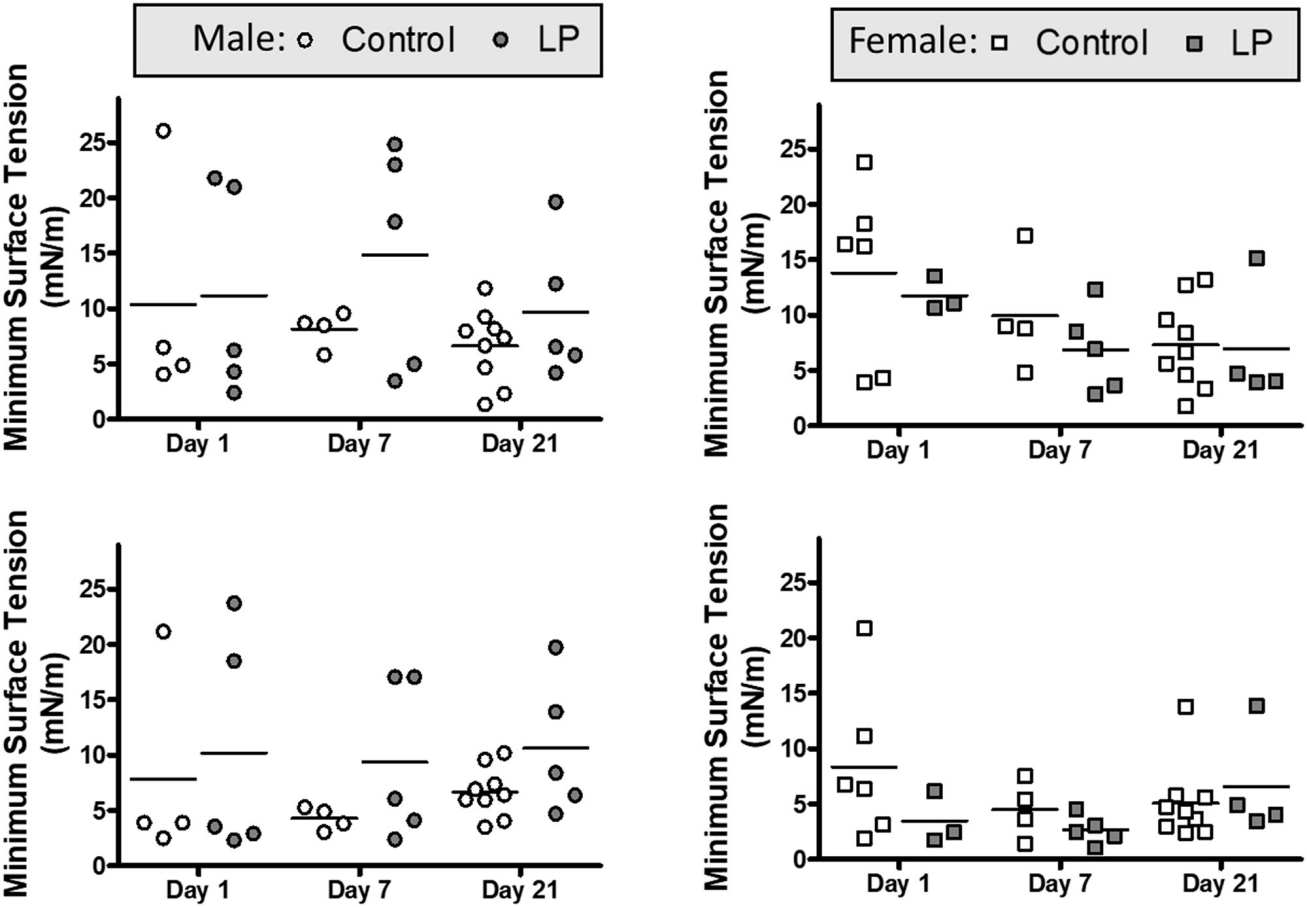
Surfactant biophysical activity. Minimum surface tensions after 1 and 10 compression/expansion cycles of surfactant LA from Control and LP males (A, C) and females (B, D) postnatal d1, d7 and d21, as analyzed on the constrained drop surfactometer.

To further analyze the surfactant system, the biophysical properties of isolated LA were examined on the CDS. The data (Fig 5) showed that the minimum surface tension obtained by the isolated surfactant was not significantly different among all groups regardless of age, diet or sex. Further analysis of the biophysical analysis data, Table 2, showed that samples from all groups were compressed to a similar extend, and were able to reach similar surface tensions during adsorption, and during expansion (maximum surface tension).

**Table 2:** Surfactant equilibrium surface tension upon adsorption, area compression ratios and maximum surface tensions at cycle 10 for control and LP rats at d1, d7, and d21.

	Age	Male		Female	
		Control diet	LP diet	Control diet	LP diet
**Surface Tension Following Adsorption (mN/m)**	**d1**	25.2 ± 1.3	26.0 ± 0.6	25.2 ± 0.3	26.0 ± 0.6
	**d7**	24.1 ± 1.0	25.1 ± 2.1	24.8 ± 1.2	24.7 ± 0.5
	**d21**	23.8 ± 0.4	23.5 ± 0.4	23.7 ± 0.5	23.4 ± 1.1
**% of the Original Area** **at Min Surface Tension** **(Cycle 10)**	**d1**	72.0 ± 2.2	74.4 ± 4.1	71.4 ± 2.2	69.2 ± 1.8
	**d7**	70.1 ± 1.6	71.8 ± 5.0	74.1 ± 3.3	72.7 ± 2.4
	**d21**	71.1 ± 0.9	71.5 ± 1.0	70.1 ± 0.6	73.4 ± 1.6
**Max Surface Tension** **(mN/m) (Cycle 10)**	**d1**	36.8 ± 2.1	38.2 ± 1.1	41.5 ± 1.5	40.9 ± 0.1
	**d7**	40.7 ± 0.9	44.4 ± 1.4	36.3 ± 2.2	41.5 ± 1.2
	**d21**	36.4 ± 1.2	36.2 ± 1.1	37.9 ± 1.8	33.7 ± 2.0

## Discussion

This study tested the hypothesis that FGR induced by maternal protein restriction during perinatal life alters lung mechanics and the surfactant system. The results supported this hypothesis with regards to lung mechanics as significantly lower lung compliance, higher tissue elastance and higher airway resistance were observed in LP female offspring at day 21. In addition, female LP offspring exhibited lower surfactant pool sizes at day 1, which was not observed in the male offspring. No differences in surfactant pools on other postnatal days were observed and surfactant biophysical properties were not significantly different among groups at any of the timepoints. Collectively, this data suggests that FGR has a different impact on pulmonary function in female, as compared to male, offspring. It also indicates that the significant alterations to lung compliance at day 21 in female offspring occurs in the absence of alterations to the pulmonary surfactant system.

To address our hypothesis we used an established model of FGR induced by maternal protein restriction during gestation and lactation. Unlike maternal hypoxia or undernutrition, this isocaloric diet has no impact on maternal food intake or weight gain, but leads to placental insufficiency resulting in relatively low bodyweight in both male and female offspring at birth as well as during the postnatal period [19,28]. This is a relevant model given that, in humans, protein (and amino acid) deficiency underlies fetal growth restriction in idiopathic IUGR, which represents 8% of pregnancies [9,29]. Protein deficiency in the fetus can result from placental insufficiency or a poor maternal diet [9,29]. Moreover, fetal protein deficiency also occurs in preeclamptic pregnancies [30,31]. We combined this relevant model with the analysis of lung mechanics utilizing the FlexiVent, and examination of the alveolar surfactant pools [22,32]. Moreover, we also took advantage of the CDS, which allows accurate surface tension measurement on small samples, to analyze the biophysical activity of surfactant from individual neonatal rats [27].

Our main observation was the lower lung compliance in female LP offspring compared to the control diet, whereas compliance was not altered in the male LP offspring. A similar pattern of a female specific change in lung compliance in a maternal calorie restriction model of FGR in mice [33]. These changes were observed at 4 and 12 weeks of age, indicating that the change in compliance may be maintained post-weaning. Further, a change in compliance, as well as other respiratory parameters, was also observed in a sheep model of FGR, however sex-differences were not examined [13]. As lung compliance represent the combined properties of tissue elasticity and surfactant biophysics, our data further enhances the previously published data by demonstrating that altered surfactant pools or activity were not responsible for the observed change in compliance in female animals. It is therefore likely that structural alterations contribute to this pulmonary FGR phenotype [14]. Changes in the structural components that directly contribute to compliance such collagen and elastin were not examined; this requires further investigation.

In addition to a female specific difference in compliance, we also observed a change in surfactant pools, both total and the surfactant subfractions, in female animals with the LP diet at neonatal day 1. Together these observations indicate a clear sexual dimorphism within the pulmonary response to FGR. Although the objective of the current study was not to understand the underlying pathways leading to these sex differences, previous studies in a variety of organs implicate a variety of mechanism including epigenetics, hormonal regulation and the timing of organ development [4]. For example, in a series of studies by Adamson and King it was observed that alveolar type II cell differentiation and surfactant synthesis occurred earlier in female rats as compared to males and that this process was affected by sex hormones [34–36]. In our study these effects may have led to the different surfactant pool sizes at postnatal day 1 under the condition of growth restriction.

The lack of differences in surfactant, other than the observed effects at day 1, was somewhat surprising in view of previous publications. Analysis of the steady-state mRNA levels of the surfactant proteins were reported to be decreased both a calorie restriction and a hypoxia model of FGR in the rat, as well as in a placental restriction model in sheep [14,17,18]. These studies did not report specific sex differences. It is feasible that the specific experimental model, the specific outcomes, and the timing of the fetal insult could have influenced the lack of differences in our experiments. In the aforementioned studies, the fetal insults leading to FGR were initiated during the latter stages of gestation, which is different from our model in which maternal protein was restricted for the entire gestational and lactational period [14,16,18]. Further, nutrition restriction or hypoxia may have differential effect on lung development given both maternal food intake and maternal weight gain are diminished in these models of FGR, impacting both the mother and fetus [37].

One of the interesting findings was the progression of surfactant pool sizes during the neonatal period. Surfactant analysis of the different age groups showed relatively higher levels of weight corrected, total surfactant, LA and SA pool sizes at day 1 compared to the later ages. In a previous studies, relative large surfactant pool sizes were also observed at birth in healthy offspring and were thought to be required for the adaptation to air-breathing [38,39]. A second observation was that the surfactant was at d21 contained a higher percentage of LA as compared to the earlier days. Specifically, within the first 24 hours after birth, surfactant appears to switch from mostly being in the LA form, to higher amounts of the SA subfraction [38]. As the change in surface area is one of the main mechanisms by which LA convert into SA, the breathing pattern during the postnatal period results in an equilibrium in the percentage of LA due to the secretion of LA, the conversion of LA into SA and the reuptake of SA [40,41]. Over time, as the lung develops and breathing pattern changes, this equilibrium will switch to adult levels [23]. Overall, our results supported these previous concepts and expand upon them by first, showing that the same regulating mechanism to maintain high surfactant pool sizes existed in both sexes, and second, demonstrating that these mechanisms were conserved in LP newborn males and females.

One of the main rationales for studying FGR in animal models is the translation to clinical conditions in humans and clinical implication later in life [1,9]. Although further studies are warranted there is epidemiological evidence that FGR is associated with decreased pulmonary health in general and that it is linked to specific pulmonary complications such as asthma [7]. More importantly, many lung conditions are related to either direct, such as pulmonary exposure to toxins, particles and organisms [42–44], or indirect, such as sepsis [45], insults to the lung. Altered responses to these pulmonary insults could impact the clinical outcomes in FGR offspring as compared to controls. Although this concept requires further experimentation, enhanced susceptibility of the lung to pulmonary insults has been reported in other conditions such as alcoholism, and old age [46,47]. Thus, we speculate that the observed changes in lung mechanics in fetal FGR offspring may provide an enhanced susceptibility to lung injuries.

It should be noted that our study included several limitations. As a technical limitation of the utilized equipment, we were unable to measure lung mechanics in the postnatal day 1 animals. Similarly, for the two other postnatal days, animals underwent analysis of lung mechanics prior to lavage and surfactant: it is feasible that those lung perturbations had an impact on the lung and the surfactant system. Further, as one of our objectives was to evaluate surfactant biophysical function, lungs of all animals were lavaged which compromises the use of the lung tissue for additional measurements such as lung structural assessments. Although the isocaloric protein restriction model of FGR has been utilized extensively, it should be acknowledged that no single animal model represents all features of all individual human low birthweight babies [19,20]. Multiple approaches with various animal models combined with clinical studies are required to provide a more complete picture of this complex clinical condition with its long and short-term consequences.

Overall, we conclude that female offspring are differentially affected by a maternal low protein diet as compared to males. Pulmonary surfactant levels at birth and lung compliance at postnatal day 21 were significantly lower in female, but not in male, low protein offspring. The decrease in compliance at day 21 is not associated with changes in surfactant activity and are therefore likely related to structural features of the lung. Further studies are required to test if the observed differences in female offspring translate into an altered susceptibility to pulmonary insults. Moreover, given the lung is still developing in neonatal life and is subject to alterations by nutritional cues during this period, it remains plausible that safe pharmaceutical or dietary interventions could reverse the detrimental effects on lung function, both short- and long-term.
